# Better survival with lobectomy versus sublobar resection in patients with hypermetabolic c-stage IA lung cancer on positron emission tomography/computed tomography

**DOI:** 10.1093/ejcts/ezae347

**Published:** 2024-09-25

**Authors:** Satoshi Shiono, Makoto Endo, Hikaru Watanabe, Satoshi Takamori, Jun Suzuki

**Affiliations:** Department of Surgery II, Faculty of Medicine, Yamagata University, Yamagata, Japan; Department of Thoracic Surgery, Yamagata Prefectural Central Hospital, Yamagata, Japan; Department of Surgery II, Faculty of Medicine, Yamagata University, Yamagata, Japan; Department of Thoracic Surgery, Yamagata Prefectural Central Hospital, Yamagata, Japan; Department of Surgery II, Faculty of Medicine, Yamagata University, Yamagata, Japan

**Keywords:** Positron emission tomography/computed tomography, Lobectomy, Sublobar resection, Thoracic surgery

## Abstract

**OBJECTIVES:**

The clinical trial showed that sublobar resection was not inferior to lobectomy in terms of disease-free survival in patients with peripherally located non-small-cell lung cancer ≤2 cm. However, it is not clear whether sublobar resection is indicated for all types of c-stage IA lung cancer. The purpose of this study was to clarify whether sublobar resection is indicated for c-stage IA hypermetabolic lung cancer.

**METHODS:**

Patients with c-stage IA lung cancer who underwent F-18 fluorodeoxyglucose positron emission tomography/computed tomography and lobectomy or sublobar resection were assessed. Of these, patients who had a maximum standardized uptake value ≥3.0 on positron emission tomography/computed tomography were evaluated. We compared survival rates after lobectomy versus sublobar resection. Propensity score matching was performed to balance patient characteristics between groups.

**RESULTS:**

Between April 2004 and March 2023, 723 patients underwent lobectomy or sublobar resection and had a maximum standardized uptake value ≥3.0 on positron emission tomography/computed tomography. Lobectomy and sublobar resection were performed in 532 (73.6%) and 191 (26.4%) patients, respectively. Both the 5-year overall and disease-free survival rates were worse after sublobar resection compared with lobectomy (62.3% vs 79.9% and 53.9% vs 70.3%, respectively). After propensity score matching, the 5-year overall and disease-free survival rates remained worse after sublobar resection compared with lobectomy (60.7% vs 75.2% and 51.6% vs 67.7%, respectively).

**CONCLUSIONS:**

Patients with c-stage IA hypermetabolic lung cancer with standardized uptake value ≥3.0 on positron emission tomography/computed tomography had a worse prognosis after sublobar resection than after lobectomy.

## Graphical Abstract

**Figure:**
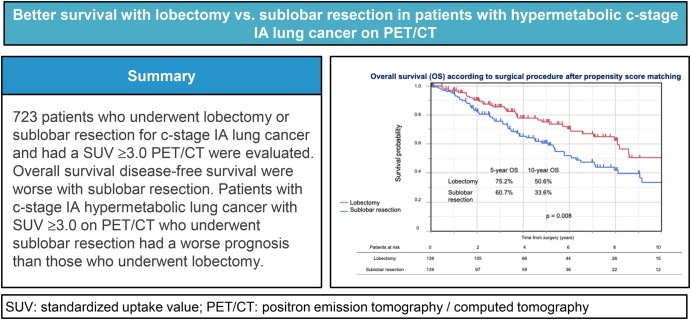

## INTRODUCTION

Lung cancer remains the leading cause of death worldwide, and 1 796 144 patients died from lung cancer in 2020 [1]. However, early-stage lung cancers classified as cT-1aN0M0 according to the TNM classification have better survival and can be cured by surgery. The CALGB 140503 trial showed that sublobar resection, which includes segmentectomy and wedge resection, is not inferior to lobectomy in terms of disease-free survival (DFS) in patients with peripherally located non-small-cell lung cancer (NSCLC) ≤2 cm. It also revealed that overall survival (OS) was similar after sublobar resection versus lobectomy [2]. The JCOG0802/WJOG4607L trial showed superiority of segmentectomy over lobectomy in terms of OS for early-stage NSCLC. Relapse-free survival was similar after both procedures [3]. On the other hand, there are some concerns with executing sublobar resection. In the JCOG trial, the incidence rate of loco-regional recurrence was significantly higher after segmentectomy (11%) than after lobectomy (5%) [3]. Considering the results of that trial, preoperative evaluation and an accurate surgical procedure are required for sublobar resection, and some patients may not be suitable for sublobar resection.

18F-fluorodeoxyglucose positron emission tomography (PET)/computed tomography (CT) provides information on lung cancer staging in the preoperative setting. 18F-fluorodeoxyglucose uptake, which is quantified as the standardized uptake value (SUV), is reported to be a predictive marker of biologically aggressive behaviour in lung cancers [4–6]. Lung cancers with a higher SUV are regarded as hypermetabolic. Due to their highly aggressive characteristics, sublobar resection could not be indicated. Thus, the efficacy of sublobar resection for hypermetabolic c-stage IA lung cancer should be clarified.

We hypothesized that lobectomy confers better survival for patients with hypermetabolic c-stage IA lung cancer. The purpose of this study was to clarify whether sublobar resection is indicated for c-stage IA hypermetabolic lung cancer.

## PATIENTS AND METHODS

This study was conducted in accordance with the principles of the Declaration of Helsinki. The institutional ethics committee approved this study and waived the need for informed consent (approval no. 2023-320).

### Study design

This retrospective study was undertaken using a database of patients prospectively collected from 2 institutions. This database contains the following data: (i) preoperative patient demographics, (ii) the results of radiological findings, including chest CT and PET/CT, (iii) stage, (iv) surgical procedures, (v) pathological findings, (vi) postoperative therapy and (vii) outcomes. Since the lung cancer TNM staging system is revised almost every 10 years, it changed from the 6th to 8th editions during the study period. The TNM stage of patients who underwent surgery before 2017 was reclassified according to the TNM 8th edition [7].

Between May 2004 and March 2023, 1958 consecutive patients underwent preoperative PET/CT and complete resection for c-stage IA lung cancer. Based on a previous study [8], we defined a hypermetabolic tumour as lung cancer with an SUV ≥3.0 on PET/CT. The exclusion criteria were as follows: multiple lung cancers, superior sulcus tumour, early hilar lung cancer, pneumonectomy, bronchial resection, neoadjuvant therapy and unavailable data.

### Preoperative assessment

Preoperative biopsy was not mandatory. If a preoperative pathological diagnosis was not made, intraoperative frozen sections were used for diagnosis.

PET/CT was performed before surgery to detect solid lung nodules at 2 institutions and the protocol have been previously described [9].The SUVmax was measured in the region of interest, which was established by the radiologists who evaluated the PET scan, and was then automatically calculated using software.

### Surgery

Lobectomy and sublobar resection performed via open thoracotomy or a thoracoscopic approach. Sublobar resection was performed in patients with nodules with subsolid density, poor performance status or impaired respiratory function and/or severe comorbidities, and in those who requested it. Segmentectomy was performed in patients who were enrolled in the clinical trial [3] or those with centrally located subsolid nodules. Mediastinal lymphadenectomy was performed in patients undergoing lobectomy and in a portion of sublobar resection cases. Hilar lymphadenectomy and lymph node sampling were performed in patients undergoing segmentectomy.

### Postoperative surveillance

Pathological diagnosis was based on the WHO classification [10]. During the 5 years after surgery, postoperative surveillance included physical examinations, chest X-ray or chest CT, and blood biochemical analysis were checked every 6 months. After 5 years surgery, annual chest CT was carried out until 10 years after surgery. Local recurrence was defined as tumour recurrence in the surgical margin, hilar lymph and ipsilateral mediastinal nodes, and a resected lobe. Regional recurrence was defined as pleural dissemination and the ipsilateral lung. During postoperative surveillance, if recurrence was suggested, repeat CT, PET/CT, or brain magnetic resonance imaging was performed. Diagnostic biopsy for the recurrence site was not mandatory.

We compared patients after lobectomy versus sublobar resection in terms of OS, DFS and freedom from recurrence (FFR).

### Statistical analysis

Analysis of variance was used to compare continuous variables according to surgical procedure. The chi-squared test was used to evaluate the associations between categorical variables. OS was calculated form the date of surgery to the date of death or last hospital visit. DFS was also calculated from the date of surgery to the date of recurrence detection, death of any cause or the last hospital visit. FFR was defined from the date of surgery to the date of recurrence detection, death from any cause, or last hospital visit were censored. The risk of recurrence was analysed using competing risk methods. The death without recurrence was regarded as a competing event. Patients who were alive without recurrence at the time of last hospital visit were censored. The difference in the risk of recurrence between lobectomy and sublobar resection was analysed by Gray’s test.

To control for potential differences in the background characteristics of patients who underwent lobectomy versus sublobar resection, we performed propensity scores to compare the outcomes of lobectomy and sublobar resection (1:1 matching for each group). Propensity scores were calculated using a logistic regression model and included all preoperative variables shown in Table 1 (age, sex, Charlson comorbidity index, smoking, carcinoembryonic antigen, tumour size, solid lesion size, surgery side, pulmonary function, blood gas analysis, body mass index and SUV). Propensity scores generated by the model were used for caliper matching, which used a caliper distance of 0.2 without replacement and 1-to-1 control matching. Standardized mean difference was used to assess the in the matched groups. McNemar’s test for categorical variables and paired *t* tests for continuous variables were used to analyse the patients after propensity score matching.

**Table 1:** Patient characteristics according to surgical procedure

Variables	Lobectomy, *n* = 532 (73.6%)	Sublobar resection, *n* = 191 (26.4%)	*P*
	*n* (%) or median (IQR)	*n* (%) or median (IQR)	
Age (years)	70 (64–76)	76 (70–80)	<0.001
Male/female	350 (65.8)/182 (34.2)	156 (81.7)/35 (18.3)	<0.001
Charlson comorbidity index	1 (0–2)	2 (1–3)	<0.001
Smoking index	600 (0–1000)	900 (450–1200)	<0.001
CEA (ng/ml)	3.1 (2.1–4.8)	3.4 (2.2–5.2)	0.607
Tumour size (cm)	2.2 (1.8–2.6)	1.8 (1.5–2.3)	<0.001
Solid lesion size (cm)	2.2 (1.8–2.5)	1.8 (1.5–2.2)	<0.001
Surgical side, R/L	327 (61.5)/205 (38.5)	94 (49.2)/97 (50.8)	0.004
FVC (L)	3.17 (2.61–3.75)	3.05 (2.49–3.71)	0.052
%FVC (%)	105.5 (94.7–115.6)	100.8 (88.6–115.8)	0.074
FEV1 (L)	2.28 (1.87–2.70)	2.01 (1.49–2.50)	<0.001
FEV1% (%)	73.8 (67.9–80.3)	69.2 (59.2–76.9)	<0.001
PaO_2_ (mmHg)	84.4 (77.5–92.3)	81.7 (76.0–89.5)	<0.001
PaCO_2_ (mmHg)	39.3 (36.8–41.8)	38.6 (36.1–41.5)	0.069
Body mass index	23.1 (20.8–25.4)	22.9 (20.8–25.4)	0.298
SUVmax	6.21 (4.28–9.43)	5.41 (4.02–8.81)	0.129

CEA: carcinoembryonic antigen; FEV1%: forced expiratory volume as a percentage of forced vital capacity; FEV1: forced expiratory volume in 1 s; FVC: forced vital capacity; IQR: interquartile range; SUV: standardized uptake value.

Multivariable analysis was used to identify prognostic factors for OS and DFS. All predictive factors showed in Supplementary Material, Table S1 and surgical procedures were subjected to multivariable analysis.

Data were analysed by JMP Pro software, version 16.1.0 (SAS Institute Inc., Cary, NC, USA). A *P* value <0.05 was considered to indicate statistical significance. Competing risk methods for the risk of recurrence, McNemar’s test for categorical variables and paired *t* tests for continuous variables, we used EZR version 4.2.3 (Saitama Medical Center, Jichi Medical University, Saitama, Japan).

## RESULTS

### Patient demographics

**Figure 1:**
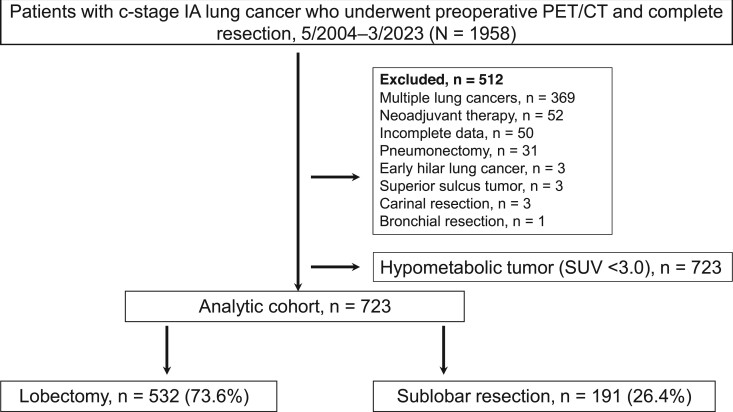
The patient enrolment schema. PET/CT: positron emission tomography/computed tomography; SUV: standardized uptake value.

Figure 1 shows the patient enrolment schema for this study. During the study period, 1958 consecutive patients underwent preoperative PET/CT and complete resection for c-stage IA lung cancer. We excluded the patients shown in Fig. 1. After further excluding patients with hypometabolic tumours (SUV < 3.0), 723 patients were included in this study. Of the 723 patients evaluated in this study, 532 (73.6%) and 191 (26.4%) underwent lobectomy and sublobar resection, respectively. In the sublobar resection group, 101 segmentectomy and 90 wedge resection cases were included. Patient characteristics including pathological findings are described in Supplementary Material, Table S1. The sublobar resection cases tended to be older males with a higher Charlson comorbidity index, higher smoking index, larger tumour size, and more impaired respiratory function compared with the lobectomy cases. There was no difference in the SUVmax between the groups (Table 1).

### Recurrence

Of the 723 patients, 161 (22.3%) developed recurrence: 109 of the 532 (20.4%) in the lobectomy group and 52 of the 191 (27.2%) in the sublobar resection group. Table 2 shows the recurrence location according to surgical procedure. The frequency of local recurrence, which included loco-regional and loco-regional with distant metastasis patterns, was 20.9% in the sublobar resection and 10.0% in the lobectomy group. Competing risk analysis shows the frequency of recurrence (Supplementary Material, Fig. S1a) (*P *=* *0.036) and local recurrence was significantly higher in the sublobar resection than lobectomy (Supplementary Material, Fig. S1b) (*P *<* *0.001). Five-year cumulative incidence of local recurrence was 22.4% in sublobar resection and 10.3% in lobectomy.

**Table 2:** Recurrence pattern according to surgical procedure

Location	Lobectomy, *n* = 532	Sublobar resection, *n* = 191	*P*
Rate of total recurrence	109 (20.4%)	52 (27.2%)	0.059
Loco-regional	42 (7.9%)	34 (17.8%)	
Distant	56 (10.5%)	12 (6.3%)	
Loco-regional + distant	11 (2.1%)	6 (3.1%)	
Local recurrence	53 (10.0%)	40 (20.9%)	<0.001

### Survival analysis

The median follow-up time from surgery was 5.2 years (range, 0–18 years). Of the 723 patients, 208 (28.8%) died: 91 (12.6%) from lung cancer, 33 (4.6%) from other cancers, 66 (9.1%) from other causes and 18 (2.5%) from unknown causes. Of the 532 lobectomy patients, 128 (24.1%) died: 62 (11.7%) from lung cancer, 18 (3.4%) from other cancers, 36 (6.8%) from other causes and 12 (2.2%) from unknown causes. Of the 191 sublobar resection patients, 80 (41.9%) died: 29 (15.2%) from lung cancer, 15 (7.9%) from other cancers, 30 (15.7%) from other causes and 6 (3.1%) from unknown causes.

**Figure 2:**
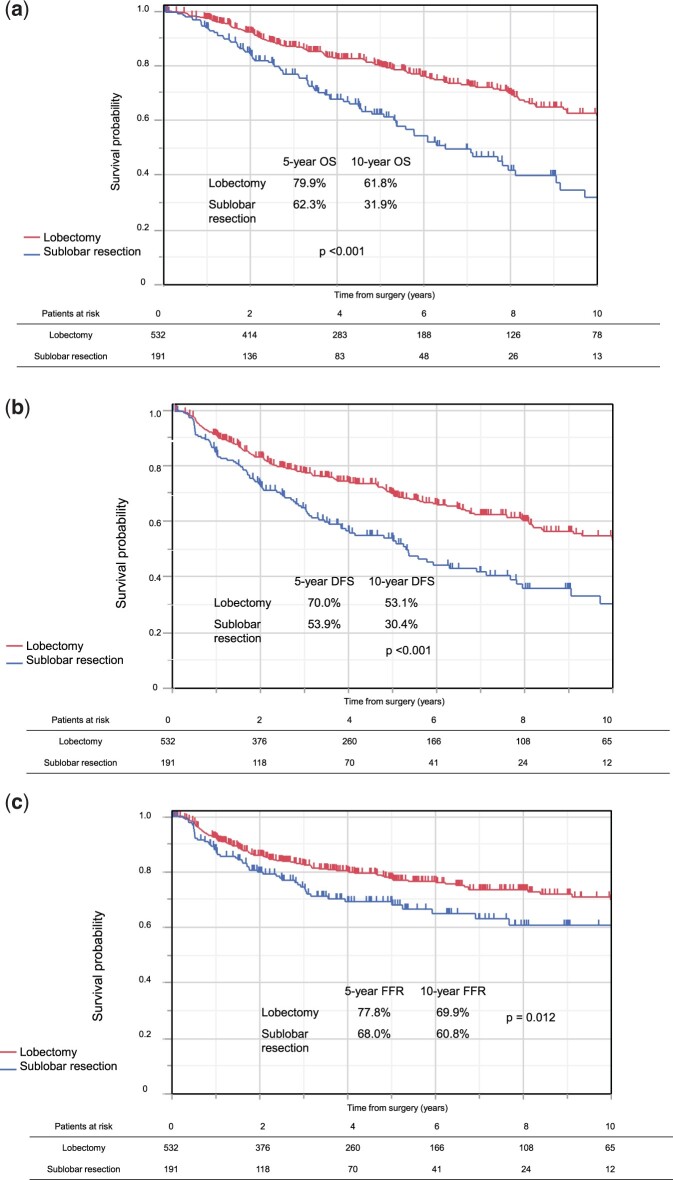
(**a**) OS according to surgical procedure in the unmatched populations. (**b**) DFS according to surgical procedure in the unmatched populations. (**c**) FFR according to surgical procedure in the unmatched populations. DFS: disease-free survival; FFR: freedom from recurrence; OS: overall survival.

In unmatched populations, OS was worse after sublobar resection than after lobectomy (*P *<* *0.001) (Fig. 2a). The 5-year OS rates were 62.3% after sublobar resection and 79.9% after lobectomy, and the 10-year OS rates were 31.9% after sublobar resection and 61.8% after lobectomy. DFS was also worse after sublobar resection than after lobectomy (*P *<* *0.001) (Fig. 2b). The 5-year DFS rates were 53.9% after sublobar resection and 70.0% after lobectomy, and the 10-year DFS rates were 30.4% after sublobar resection and 53.1% after lobectomy. FFR was significantly worse after sublobar resection than after lobectomy (*P *=* *0.012) (Fig. 2c). The 5-year FFR rates were 68.0% after sublobar resection and 77.8% after lobectomy, and the 10-year FFR rates were 60.8% after sublobar resection and 69.9% after lobectomy.

**Figure 3:**
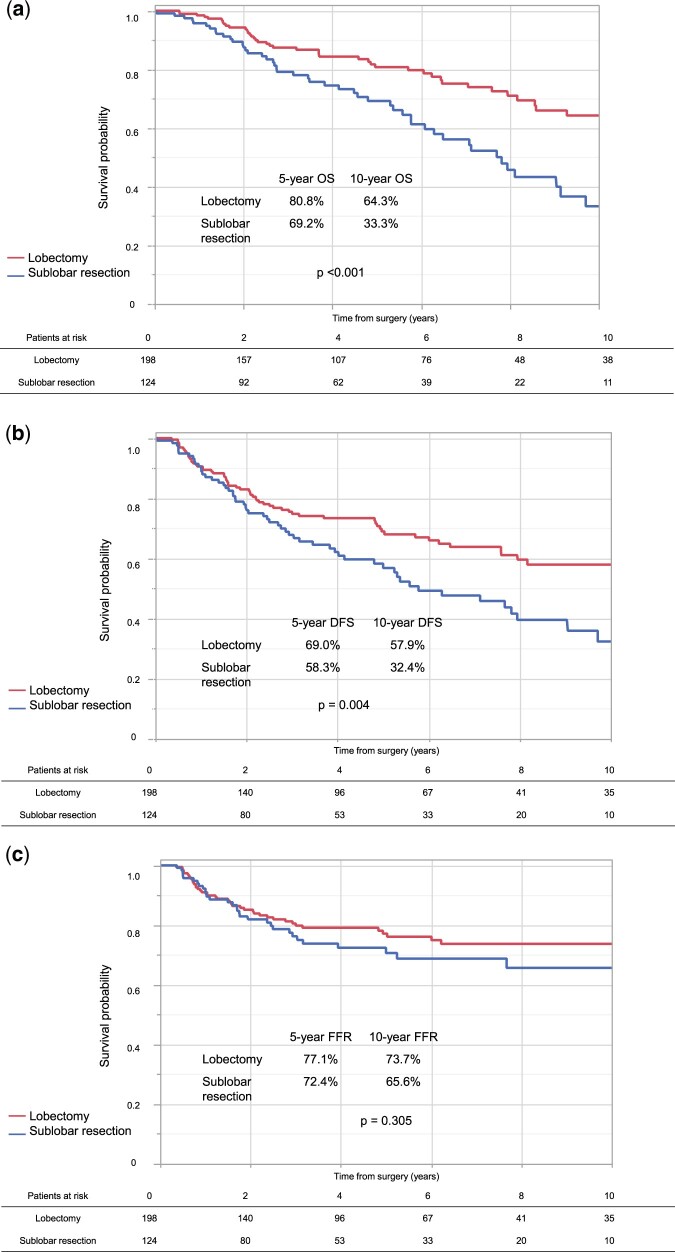
(**a**) OS according to surgical procedure in the unmatched populations of tumour size ≤2 cm. (**b**) DFS according to surgical procedure in the unmatched populations of tumour size ≤2 cm. (**c**) FFR according to surgical procedure in the unmatched populations of tumour size ≤2 cm. DFS: disease-free survival; FFR: freedom from recurrence; OS: overall survival.

Based on the results of clinical trials [2, 3], we performed subgroup analysis for the tumour of ≤2 cm and showed the results in Fig. 3. There is a similar tendency in all patients with the tumour of ≤2 cm (Fig. 3a–c).

### Survival analysis using propensity score matching

**Figure 4:**
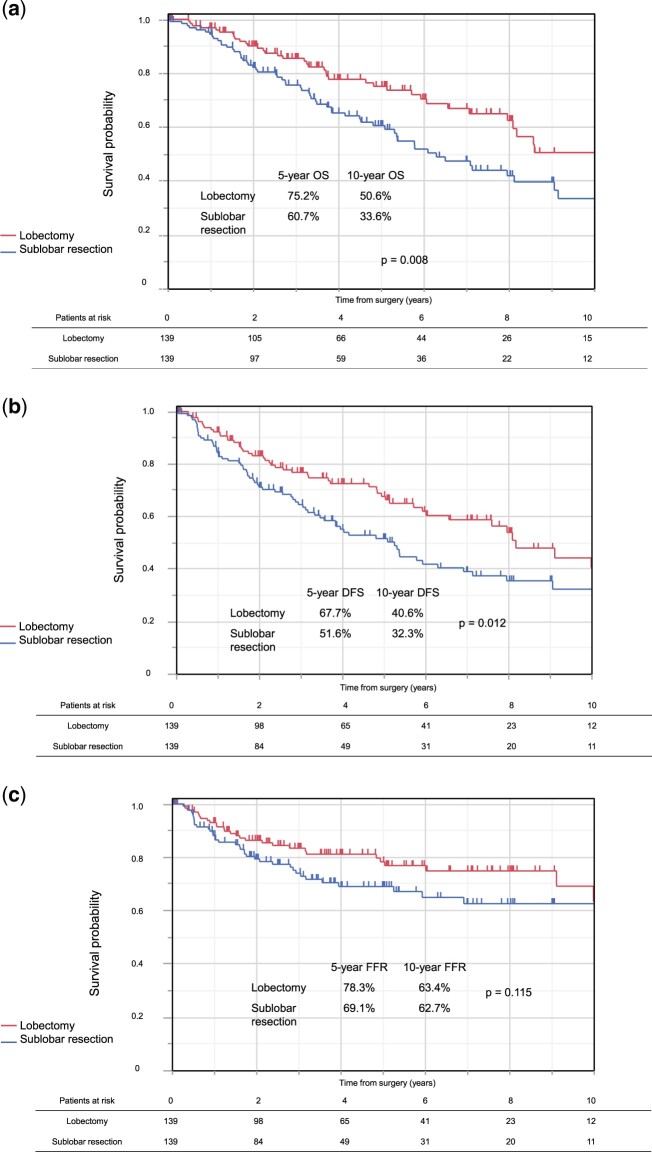
(**a**) OS according to surgical procedure in the matched populations. (**b**) DFS according to surgical procedure in the matched populations. (**c**) FFR according to surgical procedure in the matched populations. DFS: disease-free survival; FFR: freedom from recurrence; OS: overall survival.

After propensity score matching, there was no significant difference of patient characteristics between groups other than gender (Table 3). The overlaps of the propensity scores across lobectomy and sublobar resection are shown in Supplementary Material, Fig. S2. In the survival analysis, OS was worse after sublobar resection than after lobectomy (*P *=* *0.008) (Fig. 4a), with 5-year rates of 60.7% and 75.2% and 10-year rates of 33.6% and 50.6%, respectively. DFS was also worse after sublobar resection than after lobectomy (*P *=* *0.012) (Fig. 4b), with 5-year rates of 51.6% and 67.7% and 10-year rates of 32.3% and 40.6%, respectively. Regarding FFR, we did not detect a significant difference between the surgical procedures (Fig. 4c), although FFR was slightly lower after sublobar resection than after lobectomy. The rate of local recurrence was significantly higher in the sublobar resection cohort after propensity score matching (Supplementary Material, Table S2). We performed competing risk methods to assess the risk of recurrence, while there was no significant difference in recurrence (*P *=* *0.177) (Supplementary Material, Fig. S3a), local recurrence was still higher in sublobar resection cohort (*P *=* *0.007) even after propensity score matching (Supplementary Material, Fig. S3b).

**Table 3:** Patient characteristics according to surgical procedure after propensity score matching

Variables	Lobectomy, *n* = 139 (50.0%)	Sublobar resection, *n* = 139 (50.0%)	*P*	SMD
	*n* (%) or median (IQR)	*n* (%) or median (IQR)		
Age (years)	75 (71–78)	74 (69–80)	0.567	0.068
Male/female	113 (81.3)/26 (18.7)	108 (77.7)/31 (22.3)	<0.001	0.075
Charlson comorbidity index	2 (1–2)	2 (0–2)	0.277	0.126
Smoking index	880 (300–1100)	800 (400–1100)	0.732	0.037
CEA (ng/ml)	3.5 (2.3–5.0)	3.4 (2.1–6.0)	0.382	0.105
Tumour size (cm)	2.0 (1.7–2.4)	1.9 (1.5–2.5)	0.410	0.056
Solid lesion size (cm)	2.0 (1.5–2.4)	1.9 (1.5–2.4)	0.461	0.057
Surgical side, R/L	73 (52.5)/66 (47.5)	73 (52.5)/66 (47.5)	0.611	0.000
FVC (L)	3.16 (2.60–3.57)	3.15 (2.51–3.81)	0.953	0.003
%FVC (%)	104.6 (90.6–116.0)	104.3 (90.0–118.2)	0.926	0.005
FEV1 (L)	2.16 (1.77–2.46)	2.18 (1.59–2.58)	0.746	0.028
FEV1% (%)	69.9 (64.5–75.3)	72.0 (64.5–77.7)	0.815	0.027
PaO_2_ (mmHg)	81.8 (76.3–89.7)	82.5 (76.1–91.0)	0.963	0.001
PaCO_2_ (mmHg)	38.1 (35.9–40.6)	38.5 (36.3–41.6)	0.381	0.104
Body mass index	22.9 (20.7–25.6)	23.3 (20.8–25.5)	0.776	0.037
SUVmax	6.20 (4.28–8.33)	5.44 (4.04–8.88)	0.531	0.074

CEA: carcinoembryonic antigen; FEV1%: forced expiratory volume as a percentage of forced vital capacity; FEV1: forced expiratory volume in 1 s; FVC: forced vital capacity; IQR: interquartile range; SMD: standardized mean difference; SUV: standardized uptake value.

### Multivariable analysis

Multivariable analysis was performed to explore the prognostic significance of sublobar resection. Sublobar resection was identified as a significant risk factor for worse prognosis in terms of OS and DFS (Supplementary Material, Tables S3 and S4).

## DISCUSSION

Randomized clinical trials have revealed strong evidence of the effectiveness of sublobar resection for early-stage lung cancers [2, 3]. Sublobar resection may be a reasonable approach for early-stage NSCLC. On the other hand, there is some controversy regarding the indication of sublobar resection for some groups of clinical stage IA NSCLC patients. Several retrospective analyses showed inferiority of segmentectomy for larger-sized NSCLC tumours [11, 12], whereas other studies suggested that the indication for segmentectomy can be expanded to clinical T1cN0M0 NSCLC [13, 14]. In a Japanese real-world nationwide database study, OS and DFS were worse after segmentectomy than after lobectomy in cT1c patients [12]. Expanding of the indication of sublobar resection for cT1c lung cancers should be carefully considered. In terms of a sufficient surgical margin and better detection of lymph node metastasis [15, 16], lobectomy is superior to sublobar resection for larger-sized early lung cancers. A *post hoc* supplemental analysis of the JCOG data was conducted for small-sized NSCLC with radiologically pure-solid density on thin-section CT, which is assumed to have aggressive features. The results revealed that younger females tended to have better relapse-free survival after lobectomy than after segmentectomy [17].

Multiple studies have shown that hypermetabolic lung cancers detected by PET/CT are more likely to have aggressive biological behaviour and a tendency to recur [4–6]. Our group also suggested that preoperative PET/CT and thin-section CT findings are useful for detecting aggressive lung adenocarcinoma [9]. Thus, there is a concern with performing sublobar resection for hypermetabolic c-stage IA lung cancers. In the present study, we used the SUV measured by PET/CT as a factor to evaluate hypermetabolic activity. However, because the SUV is affected by various parameters, the use of SUV to predict recurrence and survival is controversial and has limitations [18]. A review reported that the threshold SUV used by previous studies in univariable analyses ranged from 3.3 to 20 [5]. Kamigaichi *et al.* [8] investigated radiologically aggressive c-stage IA1 and 2 lung cancers and reported an SUVmax of 3.0 as the median value of aggressive lung cancer; we used this value as a threshold.

Our results after propensity score matching suggest that sublobar resection for hypermetabolic c-stage IA lung cancer tends to confer worse OS and DFS. Before and after propensity score matching, the local recurrence rate was higher after sublobar resection than after lobectomy (Table 2, Supplementary Material, Table S2 and Fig. S3b). We suggest that preoperative radiological evaluation using PET/CT helps surgeons determine the best surgical procedure. According to a recent study from the Mayo Clinic, a higher SUV is significantly related to pN1 upstaging of c-stage IA NSCLC [19]. While the patients who underwent sublobar resection seemed to have a worse general condition and impaired respiratory function, pathological characteristics of lobectomy group included larger tumour sizes, higher rate of lymph node metastasis, and lymphovascular and pleural invasions, even after propensity score matching (Supplementary Material, Tables S5 and S6). Given that invasive lung cancers were much more prevalent in the lobectomy group, lobectomy may provide longer survival for patients with hypermetabolic lung cancers.

Since wedge resection could not perform a systematic lymph node dissection and obtain a sufficient surgical margin, segmentectomy is thought to be superior to wedge resection in terms of surgical curability [20]. However, a retrospective analysis compared lobectomy and sublobar resection using propensity score matching [15]. The CALGB140503 trial defined sublobar resection as both wedge resection and segmentectomy [2]. In a subset analysis of that trial, the difference in surgical outcome among lobectomy, segmentectomy, and wedge resection was investigated, and the results revealed no differences in DFS, OS, or lung cancer-specific survival among the surgical procedures [21]. Retrospective studies showed a similar result [22]. According to a recent report, nearly 20% of reported segmentectomies do not fit the criteria of true segmentectomy [23]. Considering the papers, we determined that segmentectomy and wedge resection should be unified in this study. However, in this study, we performed subgroup analysis of sublobar resections. It showed that survival and recurrence rate of wedge resection was worse than segmentectomy (Supplementary Material, Fig. S4a–c). The worse outcome of sublobar resection could be affected by the outcomes of wedge resection.

We want to note the limitations of this study. First, this was an observational study of a hospital-based registry and thus involves inherent selection bias and unmeasured confounding factors. Although we used propensity score matching to reduce the differences between the lobectomy and sublobar resection groups as much as possible, some patient characteristics could not be adjusted completely. Second, while other studies have focused on ≤2 cm NSCLC [2, 3], we included 2.1–3.0 cm NSCLC cases. This could have influenced the worse prognosis and higher recurrence rate seen in the sublobar resection cases. Finally, lymph node evaluation could not be accomplished sufficiently by sublobar resection because of anatomical limitations. However, in CALGB140503 trial, negative nodes had to be confirmed intraoperatively, and mediastinal lymph nodes were removed for all patients. Insufficient lymph node evaluation could have influenced the worse OS and DFS. In addition, a part of patients with sublobar resection might be understaged and not be refereed to adjuvant treatment. It could affect the survival.

In conclusion, although there are some shortcomings regarding the study design, we found that sublobar resection conferred a worse prognosis in patients with hypermetabolic c-stage IA lung cancer detected by PET/CT after stratification by propensity score matching. Our results suggest that proper patient selection is essential before performing sublobar resection for c-stage IA lung cancer.

## Supplementary Material

ezae347_Supplementary_Data

## Data Availability

The data are not publicly available due to privacy and ethical concerns, but the data are available from the corresponding author upon reasonable request.

## Notes

Presented at the 32nd European Society for Thoracic Surgeons, Barcelona, Spain, 26–28 May 2024.

## ABBREVIATIONS

CT: Computed tomography
DFS: Disease-free survival
FFR: Freedom from recurrence
NSCLC: Non-small-cell lung cancer
OS: Overall survival
PET: Positron emission tomography
SUV: Standardized uptake value
