# Supplementary material for: Better survival with lobectomy versus sublobar resection in patients with hypermetabolic c-stage IA lung cancer on positron emission tomography/computed tomography
Source: Eur J Cardiothorac Surg. 2024 Sep 25;66(4):ezae347. doi: 10.1093/ejcts/ezae347 (PMC11460284; doi:10.1093/ejcts/ezae347)
Supplement: ezae347_Supplementary_Data [file ezae347_supplementary_data.zip › CORRECT Supplementary Tables.docx]

**Supplementary Table 1. Patient characteristics including pathological factors.**

|  | n (%) or median (IQR) |
| --- | --- |
| Surgical and preoperative factors |  |
| Surgical procedures, Lobectomy / Sublobar resection | 532 (73.6) / 191 (26.4) |
| Age (years) | 71 (37 - 91) |
| Sex, Male / Female | 506 (70.0) / 217 (30.0) |
| Charlson comorbidity index | 1 (0 – 5) |
| Smoking index | 700 (0 - 3600) |
| CEA (ng/ml) | 3.2 (0.5 – 1104.2) |
| Tumor size (cm) | 2.2 (0.6 – 7.2) |
| Solid lesion size (cm) | 2.1 (0.6 – 3.0) |
| Surgical side, R / L | 422 (58.3) / 301 (41.6) |
| FVC (L) | 3.14 (1.12 – 6.53) |
| %FVC (%) | 104.8 (51.1 – 177.4) |
| FEV1 (L) | 2.23 (0.64 – 5.30) |
| FEV1% (%) | 73.1 (28.1 – 100) |
| PaO_2_ (mmHg) | 83.9 (39.4 – 131.9) |
| PaCO_2_ (mmHg) | 39.1 (25.6 – 51.4) |
| Body mass index | 23.0 (15.2 – 34.8) |
| SUVmax | 6.05 (3.00 – 27.9) |
| Pathological factors |  |
| Pathology, Adeno / Non-Adeno | 443 (61.2) / 280 (38.7) |
| Grade, 1 / 2 / 3 / NA | 136 (18.8) / 370 (51.2) / 165 (22.8) / 52 (7.2) |
| Total tumor size (cm) | 2.1 (0.4 – 6.0) |
| Size of invasive focus (cm) | 1.8 (0 – 5.5) |
| Lymph node metastasis, N0 / N1 / N2 | 635 (87.8) / 48 (6.6) / 40 (5.5) |
| Lymphatic invasion, + / - | 79 (10.9) / 644 (89.1) |
| Vascular invasion, + / - | 110 (15.2) / 613 (84.8) |
| Pleural invasion, + / - / NA | 154 (21.3) / 567 (78.4) / 2 (0.3) |

IQR: interquartile range; CEA: carcinoembryonic antigen; FVC: forced vital capacity; FEV1: forced expiratory volume in 1 s; FEV1%: forced expiratory volume as a percentage of forced vital capacity; SUV: standardized uptake value; NA: not applicable.

**Supplementary Table 2. Recurrence pattern according to surgical procedure after propensity score matching**

| Location | Lobectomy, n = 139 | Sublobar resection, n = 139 |
| --- | --- | --- |
| Rate of total recurrence | 28 (20.1%) | 37 (26.6%) |
| Locoregional | 7 (5.0%) | 23 (16.6%) |
| Distant | 17 (12.2%) | 11 (7.9%) |
| Locoregional + Distant | 4 (2.9%) | 3 (2.2%) |
| Local recurrence | 11 (7.9%) | 26 (18.7%) |

**Supplementary Table 3. Multivariable analysis of factors associated with overall survival**

| Variables | Risk ratio (95% CI) | *p* |
| --- | --- | --- |
| Age | 1.07 (1.04 – 1.10) | <0.001 |
| Male / Female | 2.06 (1.03 – 4.13) | 0.041 |
| Charlson comorbidity index | 1.02 (0.91 – 1.14) | 0.759 |
| Smoking index | 1.00 (0.99 – 1.00) | 0.793 |
| CEA (ng/ml) | 1.00 (0.99 – 1.00) | 0.449 |
| Tumor size (cm) | 0.48 (0.16 – 1.06) | 0.125 |
| Solid lesion size (cm) | 2.58 (1.18 – 7.45) | 0.042 |
| Surgical side, R / L | 1.59 (1.18 – 2.14) | 0.002 |
| FVC (L) | 0.99 (0.99 – 1.00) | 0.907 |
| %FVC (%) | 0.28 (0.97 – 1.01) | 0.216 |
| FEV1 (L) | 1.00 (0.99 – 1.00) | 0.422 |
| FEV1% (%) | 1.02 (0.99 – 1.04) | 0.210 |
| PaO_2_ (mmHg) | 1.00 (0.98 – 1.01) | 0.689 |
| PaCO_2_ (mmHg) | 1.01 (0.97 – 1.05) | 0.554 |
| Body mass index | 0.96 (0.91 – 1.01) | 0.104 |
| SUVmax | 1.04 (1.00 – 1.07) | 0.033 |
| Sublobar resection | 1.85 (1.33 – 2.58) | <0.001 |

CEA: carcinoembryonic antigen; FVC: forced vital capacity; FEV1: forced expiratory volume in 1 s; FEV1%: forced expiratory volume as a percentage of forced vital capacity; SUV: standardized uptake value; CI: confidence interval.

**Supplementary Table 4. Multivariable analysis of factors associated with disease-free survival**

| Variables | Risk ratio (95% CI) | *p* |
| --- | --- | --- |
| Age | 1.05 (1.03 – 1.08) | <0.001 |
| Male / Female | 1.39 (0.77 – 2.50) | 0.279 |
| Charlson comorbidity index | 0.99 (0.90 – 1.10) | 0.924 |
| Smoking index | 1.00 (0.99 – 1.00) | 0.788 |
| CEA (ng/ml) | 1.00 (0.99 – 1.00) | 0.499 |
| Tumor size (cm) | 0.34 (0.14 – 0.84) | 0.019 |
| Solid lesion size (cm) | 3.64 (1.50 – 8.83) | 0.004 |
| Surgical side, R / L | 1.19 (0.92 – 1.53) | 0.190 |
| FVC (L) | 1.00 (0.99 – 1.00) | 0.841 |
| %FVC (%) | 0.99 (0.98 – 1.01) | 0.311 |
| FEV1 (L) | 1.00 (0.99 – 1.00) | 0.757 |
| FEV1% (%) | 1.02 (0.99 – 1.04) | 0.166 |
| PaO_2_ (mmHg) | 0.99 (0.99 – 1.01) | 0.878 |
| PaCO_2_ (mmHg) | 1.01 (0.97 – 1.05) | 0.643 |
| Body mass index | 0.98 (0.93 – 1.02) | 0.260 |
| SUVmax | 1.02 (0.99 – 1.05) | 0.149 |
| Sublobar resection | 1.67 (1.24 – 2.24) | <0.001 |

CEA: carcinoembryonic antigen, FVC: forced vital capacity, FEV1: forced expiratory volume in 1 s, FEV1%: forced expiratory volume as a percentage of forced vital capacity, SUV: standardized uptake value, CI: confidence interval.

**Supplementary Table 5. Pathological characteristics according to surgical procedure before propensity score matching**

| Variables | Lobectomy, n = 532 (73.6%) | Sublobar resection, n = 191 (26.4%) | *p* |
| --- | --- | --- | --- |
|  | n (%) or median (IQR) | n (%) or median (IQR) |  |
| Pathology, Adeno / Non-Adeno | 354 (66.5) / 178 (33.5) | 89 (46.6) / 102 (53.4) | <0.001 |
| Grade, 1 / 2 / 3 / NA | 98 (18.4) / 278 (52.3) /  117 (22.0) / 39 (7.3) | 38 (19.9) / 92 (48.2) / 48 (25.1) /  13 (6.8) | 0.735 |
| Total tumor size (cm) | 2.2 (1.8 – 2.6) | 1.8 (1.5 – 2.3) | <0.001 |
| Size of invasive focus (cm) | 1.8 (1.4 – 2.3) | 1.6 (1.3 – 2.0) | <0.001 |
| Lymph node metastasis,  N0 / N1 / N2 | 453 (85.2) / 42 (7.9) / 37 (7.0) | 182 (95.3) / 6 (3.1) / 3 (1.6) | <0.001 |
| Lymphatic invasion, + / - | 66 (12.4) / 466 (87.6) | 13 (6.8) / 178 (93.2) | 0.026 |
| Vascular invasion, + / - | 90 (16.9) /442 (83.1) | 20 (10.5) / 171 (89.5) | 0.028 |
| Pleural invasion, + / - | 123 (23.2) /408 (76.8) | 31 (16.3) / 159 (83.7) | 0.044 |

IQR: interquartile range; NA: not applicable

**Supplementary Table 6. Pathological characteristics according to surgical procedure after propensity score matching**

| Variables | Lobectomy, n = 139 (50.0%) | Sublobar resection, n = 139 (50.0%) | *p* | SMD |
| --- | --- | --- | --- | --- |
|  | n (%) or median (IQR) | n (%) or median (IQR) |  |  |
| Pathology, Adeno / Non-Adeno | 76 (54.7) / 63 (45.3) | 70 (50.4) / 69 (49.6) | 0.603 | 0.087 |
| Grade, 1 / 2 / 3 / NA | 25 (18.0) / 69 (49.6) /  34 (24.5) / 11 (7.9) | 29 (20.9) / 71 (51.1) /  31 (22.3) / 8 (5.8) | 0.816 | 0.081 |
| Total tumor size (cm) | 2.0 (1.6 – 2.5) | 1.8 (1.6 – 2.5) | 0.062 | 0.263 |
| Size of invasive focus (cm) | 1.8 (1.4 – 2.4) | 1.7 (1.4 – 2.1) | 0.208 | 0.198 |
| Lymph node metastasis,  N0 / N1 / N2 | 117 (84.2) / 14 (10.1) / 8 (5.8) | 132 (95.0) / 5 (3.6) / 2 (1.4) | 0.010 | 0.361 |
| Lymphatic invasion, + / - | 15 (10.8) / 124 (89.2) | 11 (7.9) / 128 (92.1) | 0.409 | 0.099 |
| Vascular invasion, + / - | 25 (18.0) / 114 (82.0) | 14 (10.1) / 125 (89.9) | 0.056 | 0.229 |
| Pleural invasion, + / - | 34 (24.5) / 105 (75.5) | 25 (18.0) / 114 (82.0) | 0.186 | 0.159 |

IQR: interquartile range; NA: not applicable; SMD: standardized mean difference.
