# Supplementary figures and images for: Better survival with lobectomy versus sublobar resection in patients with hypermetabolic c-stage IA lung cancer on positron emission tomography/computed tomography
Source: Eur J Cardiothorac Surg. 2024 Sep 25;66(4):ezae347. doi: 10.1093/ejcts/ezae347 (PMC11460284; doi:10.1093/ejcts/ezae347)

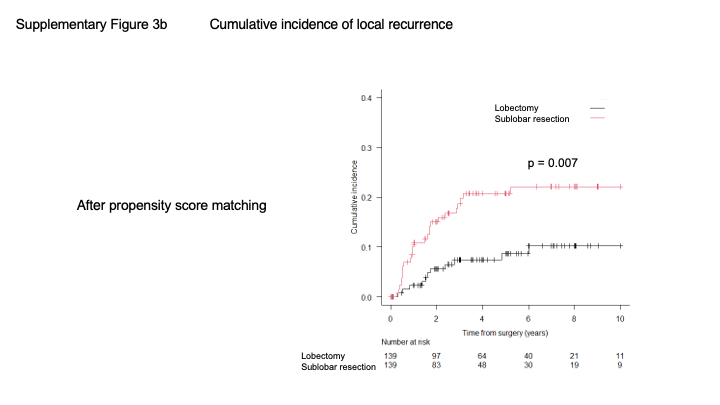

Supplement: ezae347_Supplementary_Data [file ezae347_supplementary_data.zip › suplFig3b.tiff]

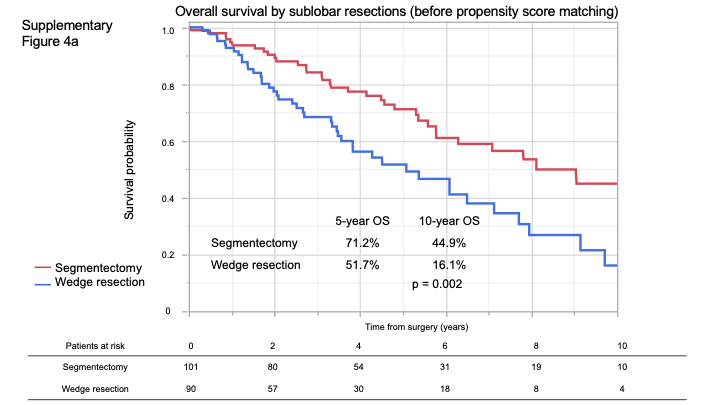

Supplement: ezae347_Supplementary_Data [file ezae347_supplementary_data.zip › suplFig4a.tiff]

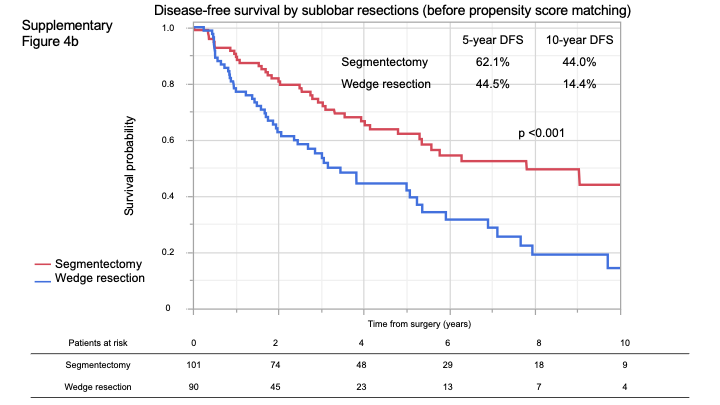

Supplement: ezae347_Supplementary_Data [file ezae347_supplementary_data.zip › suplFig4b.tiff]

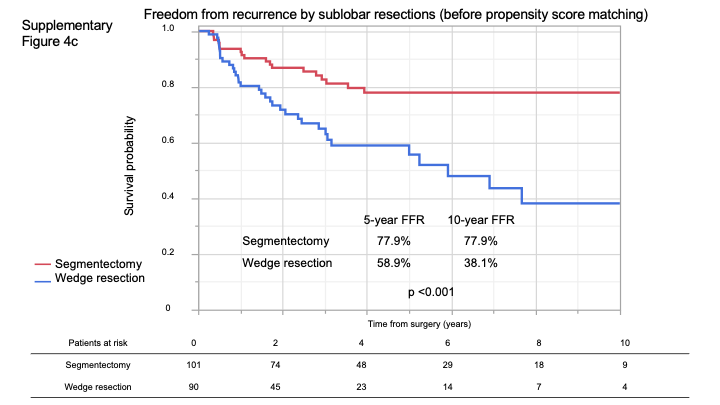

Supplement: ezae347_Supplementary_Data [file ezae347_supplementary_data.zip › suplFig4c.tiff]

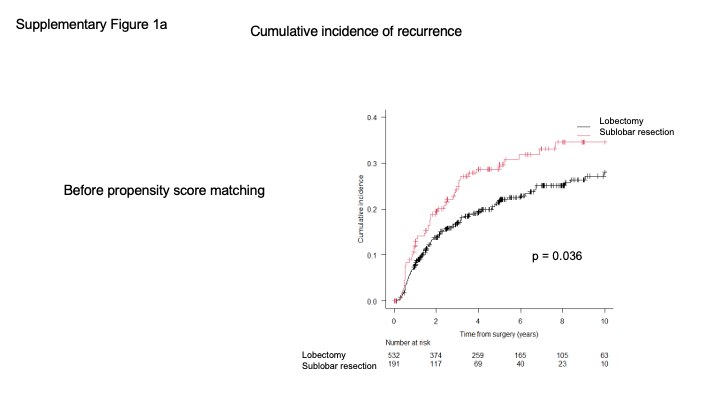

Supplement: ezae347_Supplementary_Data [file ezae347_supplementary_data.zip › suplFig1a.tiff]

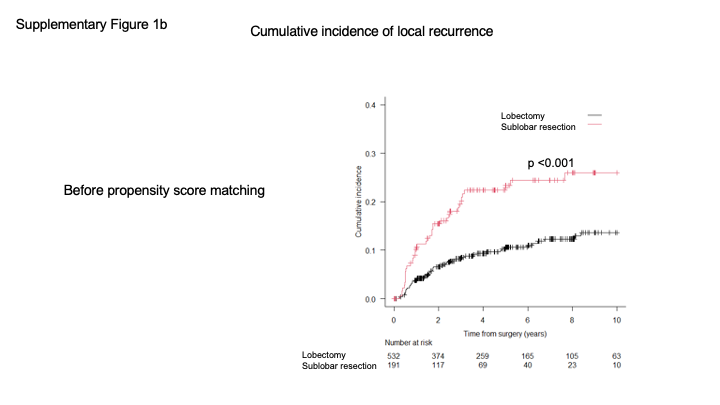

Supplement: ezae347_Supplementary_Data [file ezae347_supplementary_data.zip › suplFig1b.tiff]

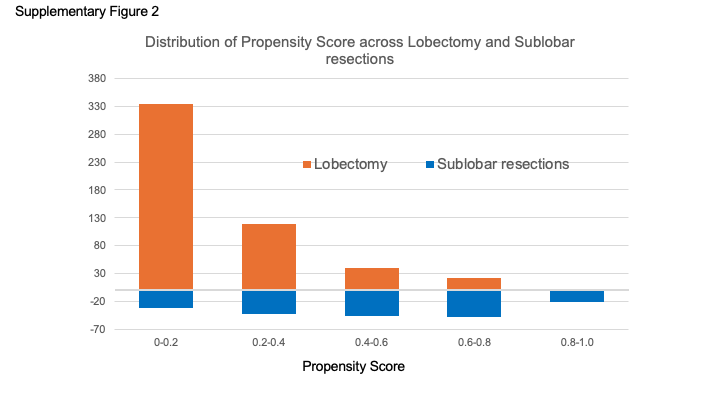

Supplement: ezae347_Supplementary_Data [file ezae347_supplementary_data.zip › suplFig2.tiff]

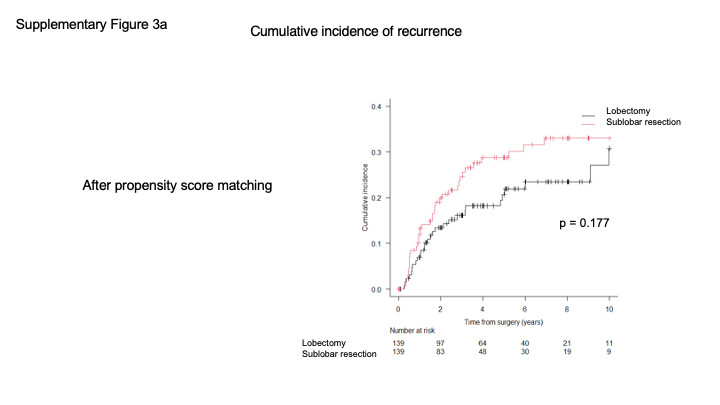

Supplement: ezae347_Supplementary_Data [file ezae347_supplementary_data.zip › suplFig3a.tiff]
